# Engineering a multicellular vascular niche to model hematopoietic cell trafficking

**DOI:** 10.1186/s13287-018-0808-2

**Published:** 2018-03-23

**Authors:** Surya S. Kotha, Brian J. Hayes, Kiet T. Phong, Meredith A. Redd, Karol Bomsztyk, Aravind Ramakrishnan, Beverly Torok-Storb, Ying Zheng

**Affiliations:** 1 Department of Bioengineering, University of Washington, Brotman Building, 850 Republican Street, Seattle, WA 98109 USA; 2 Clinical Research Division, Fred Hutchinson Cancer Research Center, Seattle, WA 98109 USA; 3 Department of Pharmacology, University of Washington, Seattle, WA 98109 USA; 4 Department of Medicine, University of Washington, Seattle, WA 98109 USA; 5 Center for Cardiovascular Biology, Institute of Stem Cell and Regenerative Medicine, University of Washington, Seattle, WA 98109 USA

## Abstract

**Background:**

The marrow microenvironment and vasculature plays a critical role in regulating hematopoietic cell recruitment, residence, and maturation. Extensive *in vitro* and *in vivo* studies have aimed to understand the marrow cell types that contribute to hematopoiesis and the stem cell environment. Nonetheless, *in vitro* models are limited by a lack of complex multicellular interactions, and cellular interactions are not easily manipulated *in vivo*. Here, we develop an engineered human vascular marrow niche to examine the three-dimensional cell interactions that direct hematopoietic cell trafficking.

**Methods:**

Using soft lithography and injection molding techniques, fully endothelialized vascular networks were fabricated in type I collagen matrix, and co-cultured under flow with embedded marrow fibroblast cells in the matrix. Marrow fibroblast (mesenchymal stem cells (MSCs), HS27a, or HS5) interactions with the endothelium were imaged via confocal microscopy and altered endothelial gene expression was analyzed with RT-PCR. Monocytes, hematopoietic progenitor cells, and leukemic cells were perfused through the network and their adhesion and migration was evaluated.

**Results:**

HS27a cells and MSCs interact directly with the vessel wall more than HS5 cells, which are not seen to make contact with the endothelial cells. In both HS27a and HS5 co-cultures, endothelial expression of junctional markers was reduced. HS27a co-cultures promote perfused monocytes to adhere and migrate within the vessel network. Hematopoietic progenitors rely on monocyte-fibroblast crosstalk to facilitate preferential recruitment within HS27a co-cultured vessels. In contrast, leukemic cells sense fibroblast differences and are recruited preferentially to HS5 and HS27a co-cultures, but monocytes are able to block this sensitivity.

**Conclusions:**

We demonstrate the use of a microvascular platform that incorporates a tunable, multicellular composition to examine differences in hematopoietic cell trafficking. Differential recruitment of hematopoietic cell types to distinct fibroblast microenvironments highlights the complexity of cell-cell interactions within the marrow. This system allows for step-wise incorporation of cellular components to reveal the dynamic spatial and temporal interactions between endothelial cells, marrow-derived fibroblasts, and hematopoietic cells that comprise the marrow vascular niche. Furthermore, this platform has potential for use in testing therapeutics and personalized medicine in both normal and disease contexts.

**Electronic supplementary material:**

The online version of this article (10.1186/s13287-018-0808-2) contains supplementary material, which is available to authorized users.

## Background

Hematopoietic cells dynamically interact with the vasculature and the surrounding microenvironment during recruitment and residence in tissues. Much effort has been made to understand the different endothelial adhesion molecules and soluble factors that regulate recruitment of roving hematopoietic cells, yet it remains unclear which niche components and surrounding stromal cells create permissive vascular environments for transmigration [1–7]. In particular, the functional contribution of stromal and endothelial phenotypes to hematopoietic recruitment within marrow vascular niche spaces is not fully understood [5, 6, 8, 9]. To date, many individual marrow components, such as mesenchymal stem cells (MSCs), macrophages, and osteoblasts, have been isolated and studied in two-dimensional *in vitro* cultures [5, 11–13]. However, since interactions are dependent on the context of a multicellular environment, more complex models are needed to recapitulate these spaces. Corresponding *in vivo* studies of the functional niche in both healthy and diseased states have been precluded by the complexity of marrow architecture and the difficulty of systematic analysis of cell behavior in dense tissue [5, 9, 10, 14, 15]. Intravital microscopy has allowed for single cell visualization of hematopoietic stem and progenitor cell (HSPC)-endothelial interactions, [6, 14, 16–20], although trafficking events are difficult to capture and the detailed dynamics of multiple niche components are still unclear. It is therefore important to develop new tools that can recapitulate multicellular microvascular environments and allow for functional analysis of hematopoietic cell trafficking.

Cell extravasation across the endothelial wall has been studied extensively for leukocytes [21–26], and HSPC trafficking has been thought to follow a similar cascade [27–31]. After vascular inflammation, the release of cytokines signal for the recruitment and arrest of leukocytes on the endothelium [21, 29, 32]. While *in vitro* and *in vivo* studies have shown that leukocytes transmigrate primarily in response to inflammatory signaling, the specifics about the cues for HSPC trafficking are not completely understood [6, 33–35]. *In vivo,* HSPCs have been shown to reside in perivascular niche spaces, composed of monocytes/macrophages, stromal fibroblasts, and proximal vasculature [5, 9, 10, 36–38]. Monocytes and monocyte-derived macrophages not only reside within these perivascular spaces, they also interact with the endothelial cells and stromal fibroblasts [10, 39, 40]. In addition, the stromal-endothelial crosstalk results in changes to the local secretion of niche-associated factors to modulate HSPC recruitment [11, 13, 36, 39, 41–43].

In the marrow, the contribution of monocytes and monocyte-derived macrophages has been noted but has not been well detailed, particularly in the context of the perivascular niche [39, 40, 44–47]. Previous studies have shown that co-culture of monocytes with marrow-derived MSCs has led to diverse outcomes due to inconsistent definition of the MSC cell type and varying co-culture conditions [4, 48, 49]. Coculture of monocytes with a defined human marrow-derived stromal fibroblast line, HS27a, in two-dimensional cultures results in close associations between the cells, changes in matrix metallopeptidase 9 (MMP9) secretion, adhesion molecule expression, cytokine secretion, and Notch signaling when compared to each cell cultured alone [44, 50, 51]. Meanwhile, co-culture of monocytes with another human marrow fibroblast line, HS5, does not change monocyte or HS5 gene expression [44, 45]. Taken together, these findings suggest that both the marrow stromal cell type and monocyte co-culture conditions must be carefully juxtaposed to understand cellular crosstalk.

In this study, we utilize a perfusable three-dimensional (3D) microvessel system to develop a marrow perivascular niche. We show that marrow-derived fibroblasts modify endothelial phenotype and the vascular microenvironment, which subsequently directs the adhesion and transmigration of perfused monocytes, CD34^+^ HSPCs, and CD34^+^ leukemic cells. We show that the circulating monocytes can enter the perivascular niche, interact with fibroblasts, and further change HSPC and leukemic cell trafficking patterns. Our study demonstrates the dynamic multicellular interactions in the marrow microenvironment, and our platform supports spatiotemporal control and monitoring of these dynamics. It also allows for the step-wise addition and subtraction of individual niche elements to further understand the hematopoietic microenvironment in health and disease.

## Methods

### Cell sourcing

#### Endothelial cells

All experiments were conducted using human umbilical vein endothelial cells (HUVECs; Lonza) between passage 4 and 6 that were grown and cultured in endothelial growth media (EBM + EGM bullet kit CC-3124, Lonza) until confluent in T-75 flasks prior to use.

#### Bone marrow fibroblast cells

Stromal fibroblast cell lines HS5-GFP and HS27a-GFP were generously provided by the Torok-Storb laboratory [51, 52]. These immortalized human marrow stromal lines were cultured in RPMI 1640 medium (Thermo Fisher Scientific) supplemented with l-glutamine (0.4 mg/mL, SAFC Biosciences), sodium pyruvate (1 mM/L), penicillin-streptomycin sulfate (100 μg/mL, Thermo Fisher Scientific), and 10% fetal bovine serum (FBS; Thermo Fisher Scientific). Stromal fibroblasts were cultured to 70% confluence in T-75 flasks and trypsinized prior to embedding in vessels. HS27a conditioned medium was removed after 5 days of culture and centrifuged prior to use in vessels for conditioned media experiments. Marrow MSCs were purchased from Lonza. MSCs were cultured in MSCGM (Lonza) in T-75 flasks and trypsinized prior to use.

#### Hematopoietic cells

Peripheral monocytes were obtained from fresh blood samples under protocols approved by the Institutional Review Board at the Fred Hutchinson Cancer Research Institute. Mononuclear cells were isolated from fresh blood through Ficoll-Paque centrifugation (specific gravity 1.077) at 200 g for 30 min at room temperature. Monocytes were isolated from this fraction through incubation with CD14 microbeads (Miltenyi Biotec) for 20 min at 4 °C, washed with phosphate-buffered saline (PBS)/2% FBS, and purified using magnetic cell sorting (Miltenyi Biotec). The monocytes were then incubated with CD14-PE and CD45-PE (BD Biosciences) for 20 min at 4 °C and washed twice with PBS/2% FBS prior to use. Healthy and acute myelogenous (patient-derived) leukemic CD34^+^ cells were purchased through the Hematopoietic Cell Processing and Repository (DK56465 and DK106829) at Fred Hutchinson Cancer Research Institute under protocols approved by the Institutional Review Board of the Fred Hutchinson Cancer Research Institute. Healthy CD34^+^ progenitor cells were isolated from granulocyte-macrophage colony-stimulating factor (GM-CSF)-mobilized HSPCs in peripheral blood and stored by the Hematopoietic Cell Processing and Repository. Healthy and leukemic CD34^+^ cells were allowed to recover overnight after thawing in StemSpan Serum-Free Expansion Medium (StemCell Technologies) supplemented with 10 ng/mL interleukin (IL)-6, 10 ng/mL stem cell factor (SCF), 10 ng/mL fms-like tyrosine kinase 3 (FLT3), 50 ng/mL thrombopoietin (TPO), and 2 U/mL erythropoietin (EPO; Peprotech). Healthy and leukemic CD34^+^ cells were stained with CD34-APC and CD45-APC (BD Biosciences) for 20 min at 4 °C and then washed twice with PBS/2% FBS prior to use.

### Vessel fabrication

**Fig. 1:**
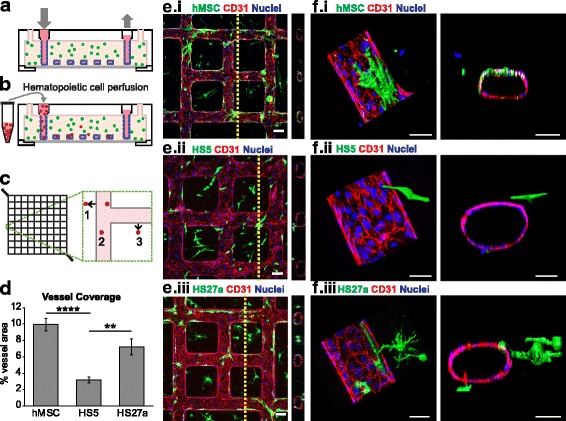
Coculture of marrow fibroblasts with engineered vessels create perfusable marrow microenvironments**. a** Schematic of microvessel platform shows a perfusable network that is then **b** perfused with hematopoietic cells. **c** Perfused hematopoietic cells are identified as migrated (1, 3) or adhered (2) within a portion of the vessel network. **d** Quantification of percent vessel coverage by human mesenchymal stem cells (hMSCs) and marrow fibroblast types show that hMSCs and HS27a cells coat vessels more than HS5 fibroblasts. ****p < 0.0001, **p < 0.005. **e** Cocultured hMSCs and marrow fibroblasts interact with endothelial vessel walls. Scale bars = 100 μm. **f** Three-dimensional reconstruction and orthogonal views of co-cultured microvessels show surface renderings of MSCs, HS5, and HS27a cells interacting with the endothelial wall. Scale bars = 50 μm

The 3D microfluidic networks were fabricated as described previously [53–55]. Briefly, soft lithography created a PDMS mold patterning a 100-μm diameter network, and injection molding over the PDMS mold created a 100-μm collagen I gel microvessel which was sealed with a collagen-coated coverslip (Fig. 1a) [53–55]. MSCs and human bone marrow-derived fibroblast cell lines HS27a and HS5 were embedded uniformly in the collagen at 1 million cells/mL. The channels were then perfused with HUVECs which adhered to the collagen and self-assembled into a functional microvessel with an open lumen. Endothelial cell culture media added to the inlet reservoir flowed through the network driven by gravity, undergoing approximately an eightfold reduction in flow (~ 0.1 dyn/cm at minimum). Vessels were cultured for 3–7 days prior to analysis.

### Hematopoietic cell perfusion through microvessels

Hematopoietic cells were perfused through vessels that had been cultured for 3–4 days. For single cell-perfused vessels, monocytes, healthy CD34^+^, or leukemic CD34^+^ cells were added to the inlet of the vessel (100,000 cells in 100 μL PBS/5% FBS) and allowed to perfuse for 30 min. Any remaining cell solution was then removed and vessels were washed with media twice for 30 min each. In double-perfused vessels, monocytes were perfused as above and then 24 h later healthy or leukemic CD34^+^ cells were added to the inlet (100,000 cells in 100 μL PBS/5% FBS) and allowed to perfuse through the vessels for 30 min (Fig. 1b). Excess cell solution was then removed and vessels were washed twice with media (30 min each). For vascular cell adhesion molecule-1 (VCAM-1) blocking experiments, a VCAM-1 blocking antibody (50 μg/mL, R&D Systems, clone BBA5) was perfused through the vessels for 1 h and vessels were briefly washed with media prior to HSPC perfusion. Then, 24 h after perfusion with cells, vessels were fixed in 3.7% formaldehyde (20 min) and washed with PBS three times (20 min each).

### Immunostaining and imaging

Prior to immunofluorescence staining, nonspecific binding was blocked with 2% bovine serum albumin (BSA)/0.5% Triton X-100 for 1 h. Staining for CD31 (Abcam), VE-cadherin (VE-cad; Abcam), von Willebrand Factor (vWF; Abcam), and α-smooth muscle actin (αSMA; Thermo Fisher Scientific) was accomplished through perfusion of immunofluorescence reagents through the microvessel network as described previously [53]. Secondary antibodies with fluorochromes Alexa Fluor 488, 567, or 647 were used. Vessels were imaged on a Nikon A1R confocal microscope.

### Scanning electron microscopy

After immunofluorescence images of microvessels were taken, microvessels were re-fixed *in situ* with 25% glutaraldehyde for 20 min and rinsed three times with PBS. The microvessels were then dissembled into top and bottom parts. The thick top portion of the collagen microvessel was dehydrated in serial ethanol washes (50%, 70%, 85%, and 100% ethanol) and further dehydrated by critical point drying (Tousimis). The vessel was then sputter coated with gold-palladium and analyzed by a FEI Sirion scanning electron microscope with an accelerating voltage of 5 kV, spot size 3.

### RT-PCR

To harvest RNA lysate from vessels, RLT Buffer was perfused through the network and collected continuously from the vessel outlet for 2 min. RNA lysate from the vessels was purified using an RNA purification kit (Qiagen). RNA purification was completed following the provided protocol and quantified using Nanodrop (Thermo Fisher Scientific). RT-PCR was performed (see Additional file 1: Table S1 for primer details) and results were normalized to RPL32 expression [56]. Significant differences were determined using Welch’s two-sample, two-tailed *t* test with Bonferroni correction (α = 0.1, *n* = 3).

### Adhesion and migration quantification

Quantification of stromal fibroblast location and hematopoietic adhesion and migration in relation to the vessel wall was analyzed using 3–10 confocal images of each vessel (*n* = 3) (Fiji, NIH). Images analyzed were selected from the low flow regions of the vessel (non-inlet or outlet regions). Image stacks of the vessel (120 μm depth) were z-projected to a single plane and coordinates of vessel borders were manually selected. Marrow fibroblast coverage of vessels is presented as a percentage of projected vessel area that is masked by fibroblasts. Coordinates of PE-labeled monocytes or APC-labeled CD34^+^ cells were located via particle analysis on thresholded images. Distances from cells to the vessel were calculated assuming that the cells migrated from the closest vessel wall (Fig. 1c). Cells that were located within the vessel boundaries were counted as adherent to the vessel wall. The distance from the nearest vessel was normalized to the vessel radius. Cell adhesion and migration data of perfused hematopoietic cells were calculated as a percent of estimated total perfused cells (based on the concentration and volume of cell suspension added to the reservoir and the gravity-driven flow rate). A sensitivity analysis of high, middle, and low estimates (75,000, 50,000, and 25,000 cells) was performed, showing no effect of the total number of perfused cells on significant differences between groups. Data are presented based on a low estimated number of perfused cells. Significant differences between groups were determined using two-sample, two-tailed student’s *t* test. Error bars represent standard error measurements.

## Results

### Stromal cells differentially interact with perfusable microvessels

To recapitulate a 3D perivascular niche *in vivo*, we engineered a 3D microvessel network in collagen gel combining lithography and injection molding processes as described previously [53–55]. The embedded lumens were seeded with human umbilical vein endothelial cells (HUVECs) to form a fully endothelialized vessel network. Three different stromal fibroblasts, namely MSCs, HS5, and HS27a cell lines, were embedded separately in the collagen gel surrounding the lumen. These co-cultured microvessel devices were maintained in culture under gravity-driven flow for up to a week. MSCs are a heterogeneous fibroblast population from the marrow and have been widely studied for their ability to interact with both the vasculature and hematopoietic cells to define a microenvironment [2, 5, 10, 57]. Here, we consider their function as stromal fibroblasts sourced from an MSC population. HS5 and HS27a are two marrow-derived stromal fibroblast cell lines that identify distinct functional phenotypes *in vitro* (see Additional file 2: Figure S1). The HS27a cell line is CD146-positive and expresses stem cell niche-associated proteins (SDF-1, angiotensin, osteopontin, and VCAM-1, among others) whereas the HS5 line (CD146^–^) secretes ample amounts of GM-CSF, G-CSF, IL-1, IL-8, MCP3, and MIP1a [51, 52]. When co-cultured with microvessels under perfusion, the three stromal fibroblasts interact differently with the endothelial cells (Fig. 1d–f). After 6 days of culture, both MSCs and HS27a cells displayed pericyte-like close association with the microvessels in that they extended processes and wrapped around the endothelium (Fig. 1d, e.i, e.ii, f.i, f.ii). In contrast, HS5 cells did not associate closely with the microvessels (Fig. 1e.iii, f.iii) but remain in the matrix. The vessel coverage was significantly increased in the MSCs and HS27a co-cultured microvessels (9.95 ± 0.76% and 7.21 ± 0.35%, respectively) over the HS5 co-cultured vessels (3.18 ± 1.0%) (Fig. 1d). Under all three conditions, the endothelium remained intact with robust junctions at regions of cell-cell contact. We therefore selected the well-defined HS27a and HS5 cell lines in this platform to represent specific marrow stromal contribution (see Additional file 2: Figure S1).

### Stromal cells modify endothelial cell phenotype

**Fig. 2:**
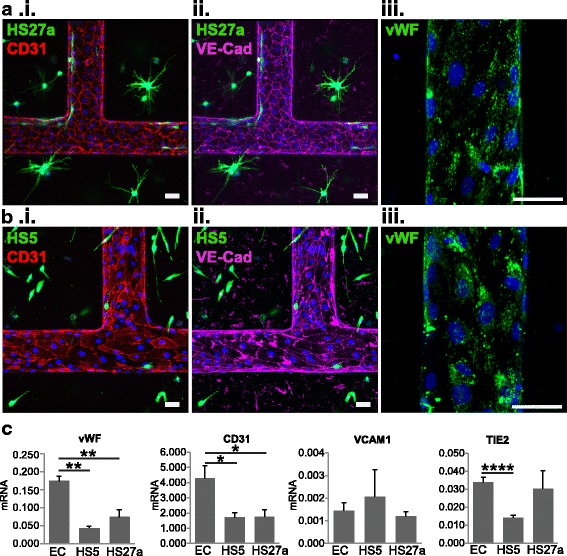
Endothelial phenotype changes with stromal cell co-culture. **a** Endothelial staining of HS27a and **b** HS5 co-cultured vessels shows differences in (i) CD31, (ii) VE-cadherin (VE-Cad), and (iii) von Willebrand Factor (vWF). **c** RNA expression within the co-culture shows differences in endothelial junctional expression and inflammatory marker expression. *p < 0.05, **p < 0.01, ****p < 0.0001. EC endothelial cell, VCAM1 vascular cell adhesion molecule-1

In addition to differences in coating, the two fibroblast cell lines around the microvessels appeared to modify endothelial phenotypes differently. Endothelial cells displayed uniform cobblestone structure in HS27a co-cultured vessels with homogeneous expression of CD31 and VE-cadherin at regions of cell-cell contact (Fig. 2a). In HS5 co-cultured microvessels, however, endothelial cells had an irregular and heterogeneous shape with varying CD31 and VE-cadherin expression and were more elongated along the direction of flow (shape index: 0.55 compared to 0.62 with HS27a co-culture; *p* < 0.05, Fig. 2a). In both stromal modified vessels, von Willebrand Factor expression is low compared to endothelial cell (EC)-only vessels, as shown by decreased appearance of Weibel Palade bodies (Fig. 2a.iii, see Additional file 3: Figure S2) [58].

RT-PCR on microvessels comparing the HS27a and HS5 stromal co-cultures to vessels with ECs alone showed a significant reduction in vWF (75% and 57% reduction in HS5 and HS27a vessels compared to EC-only vessels, respectively) and CD31 (59% and 58% reduction in expression in HS5 and HS27a vessels, respectively) expression in microvessels after co-culture. In HS5 co-cultured vessels, TIE2 levels were significantly reduced by 52% from EC-only vessels. This combination suggests an activated or inflamed endothelium when co-cultured with HS5 (Fig. 2) [59–62]. No significant change in VCAM-1 RNA expression was seen between conditions. Further expression analysis of vessels show that other inflammatory cytokines and endothelial surface markers are modified with co-culture (see Additional file 4: Figure S3). Together, the stromal fibroblasts around the microvessels modify the endothelial status and direct the formation of a specific tissue microenvironment.

**Fig. 3:**
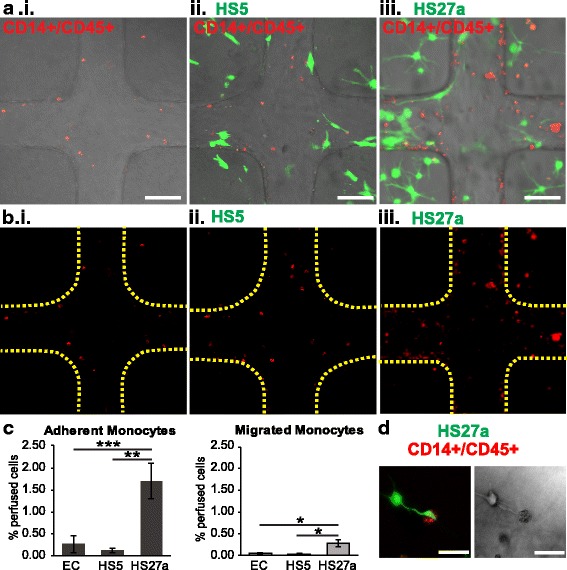
Microenvironment cues change perfused monocyte localization. **a** Monocytes (red) perfused through unmodified, HS5-, and HS27a-modified vessels adhere to the endothelium (not stained) and transmigrate into the matrix. Scale bars = 100 μm. **b** Monocytes from **a** are shown alone along with outlines of vessel walls. Scale bars = 100 μm. **c** Quantification of monocyte adhesion and migration shows the percentage of cells adhered and migrated within vessels. *p < 0.05, **p < 0.01, *** *p* < 0.001. **d** An HS27a cell wraps around a monocyte that has transmigrated into the matrix. EC endothelial cell

### Perfused monocytes adhere and transmigrate preferentially in HS27a-modified microvessels

Monocytes are known to circulate through the bloodstream and extravasate through the endothelium towards inflamed regions or tissue repair [63]. To test the functional contribution of the fibroblast-driven endothelial phenotype on monocyte interaction with vasculature, we perfused CD45^+^/CD14^+^-labeled monocytes, isolated from human peripheral blood, through the vessels and monitored their adhesion and extravasation in EC-only or co-cultured microvessels (Fig. 3). At 24 h after perfusion, the percentage of monocytes adhered to the vessel wall was significantly higher in HS27a co-cultured vessels (1.69 ± 0.40% of perfused cells) than in unmodified (0.26 ± 0.18%) or HS5 co-cultured vessels (0.11 ± 0.04%) (Fig. 3a–c). The percentage of monocytes that transmigrated into the matrix was also significantly increased in the HS27a co-cultured vessels (0.28 ± 0.07% of perfused cells) compared to EC-only (0.04 ± 0.02%) and HS5 co-cultured vessels (0.02 ± 0.01%) (Fig. 3c). In addition, some monocytes that extravasated into the HS27a-seeded matrix appear to make deliberate contact with HS27a cell projections (Fig. 3d). To examine the source of this interaction, monocyte adhesion within HS27a co-cultured vessels was compared with EC vessels with HS27a-conditioned media (see Additional file 5: Figure S4). Monocytes adhered more in HS27a co-cultured vessels than in those with HS27a-conditioned media, suggesting that contact-dependent cues rather than soluble factors modulate monocyte adhesion (see Additional file 5: Figure S4). This behavior has been seen *in vivo*, where marrow biopsy samples show the *in vivo* counterpart of HS27a cells, the CD146^+^ fibroblast, wrapped around marrow vessels and in contact with monocytes/macrophages [44, 64]. The direct interaction between monocytes and HS27a fibroblasts indicates cell-cell crosstalk for the development of a complex tissue microenvironment.

### Monocytes modify HSPC adhesion and trafficking

**Fig. 4:**
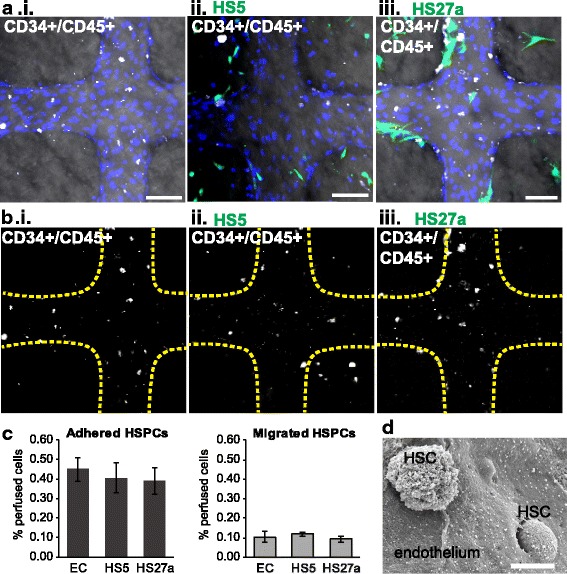
Microenvironment cues do not change CD34^+^ HSPC localization. **a** CD34^+^ HSPCs perfused through unmodified, HS5-, and HS27a-modified vessels adhere to the endothelium (not stained) and transmigrate into the matrix. Scale bars = 100 μm. **b** CD34^+^ cells from **a** are shown alone with outlines of vessel walls (yellow dotted lines). **c** Quantification of CD34^+^ HSPC adhesion and migration shows the percentage of cells adhered and migrated within vessels. **d** Scanning electron microscopic image of an hematopoietic stem cell (HSC) adhered and transmigrating through the endothelium in a vessel construct. EC endothelial cell

**Fig. 5:**
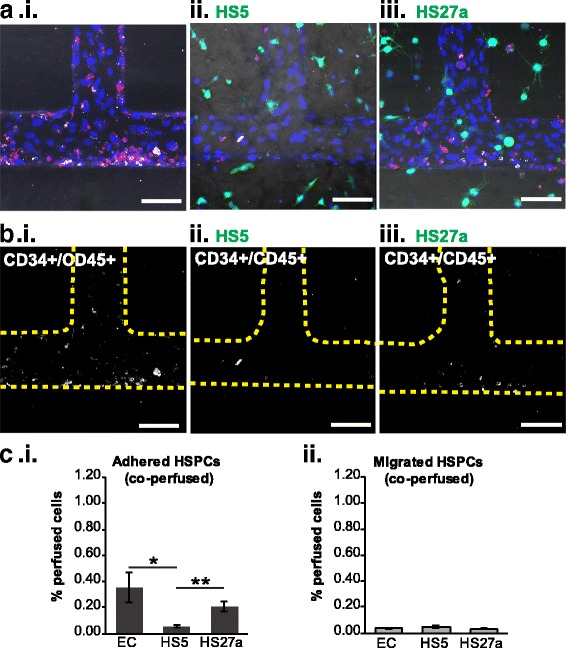
When perfused after monocytes, CD34^+^ cells preferentially adhere in endothelial cell (EC)-only and HS27a-modified vessels. **a** Healthy CD34^+^ cells (white) are perfused 24 h after monocytes (red) in each vessel type. Healthy CD34+ cells and monocytes appear to cluster together. Blue = nuclei. **b** Locations of healthy CD34^+^ cells are seen relative to vessel wall (yellow dotted line). Scale bars = 100 μm. **c** Quantification of CD34^+^ cell adhesion and migration in vessels. *p < 0.05, **p < 0.01. HSPC hematopoietic stem and progenitor cell

During tissue regeneration, HSPC recruitment may be directed by the local microenvironment. We next examined HSPC trafficking across the fibroblast-modified microvessels. Labeled CD34^+^/CD45^+^ HSPCs were perfused through the microvessel system in EC-only, HS5, and HS27a co-cultures (Fig. 4a–c). Surprisingly, we found no significant differences in adhesion (0.45 ± 0.06%, 0.40 ± 0.08%, and 0.41 ± 0.07% of perfused cells in EC-only, HS5, and HS27a vessels, respectively) or extravasation (0.10 ± 0.03%, 0.11 ± 0.01%, and 0.10 ± 0.01% for EC only, HS5, and HS27a vessels, respectively) among vessels 24 h post-perfusion. This pattern suggests that the fibroblast-endothelial microenvironments alone do not strongly influence HSPC trafficking (Fig. 4c). However, when monocytes were perfused 24 h prior to HSPCs in these same vessel co-cultures, the pattern of HSPC adhesion and extravasation was modified (Fig. 5). When monocytes were present, HSPCs preferentially adhered within the EC-only and HS27a co-cultured vessels over the HS5 co-cultured vessels (0.35 ± 0.12% and 0.20 ± 0.03% in EC-only and HS27a vessels, respectively, versus 0.05 ± 0.01% in HS5 vessels) (Fig. 5a–c).

Further analysis of data from Iwata et al. shows that the direct co-culture of monocytes with HS27a fibroblasts, but not HS5 fibroblasts or conditioned media, resulted in an overall increase in VCAM-1 expression which may partially explain the increased retention of HSPCs in HS27a vessels (see Additional file 6: Figure S5) [44]. The baseline HSPC adhesion and migration in the EC-only context did not change with the inclusion of monocytes (0.45 ± 0.06% HSPCs adhered, 0.10 ± 0.03% migrated without monocytes compared to 0.35 ± 0.12% HSPCs adhered, 0.06 ± 0.02% migrated with monocytes). In contrast, HSPC adhesion within HS5 and HS27a co-cultured vessels was reduced when monocytes were present compared with the corresponding vessels without co-perfused monocytes: adhesion was reduced from 0.40 ± 0.08% to 0.05 ± 0.01% in HS5 vessels, and from 0.39 ± 0.07% to 0.20 ± 0.04% in HS27a vessels. To explore the role of VCAM-1 in HSPC adhesion in these co-perfused vessels, we perfused a VCAM-1 blocking antibody after monocyte perfusion in the HS27a co-cultured vessels and prior to HSPC perfusion (see Additional file 7: Figure S6). However, after perfusion of monocytes, blocking VCAM-1 did not change adhesion or migration patterns of HSPCs in HS27a co-cultured vessels (see Additional file 7: Figure S6C, D). These data suggest that while monocytes and stromal fibroblasts play a role in modulating HSPC adhesion, VCAM-1 is not the adhesion molecule that significantly directs HSPC trafficking. We show that monocytes interact with stromal cells and modify the microvascular environment, which in turn changes HSPC trafficking.

### Leukemic cells show heightened response to fibroblasts without monocytes

**Fig. 6:**
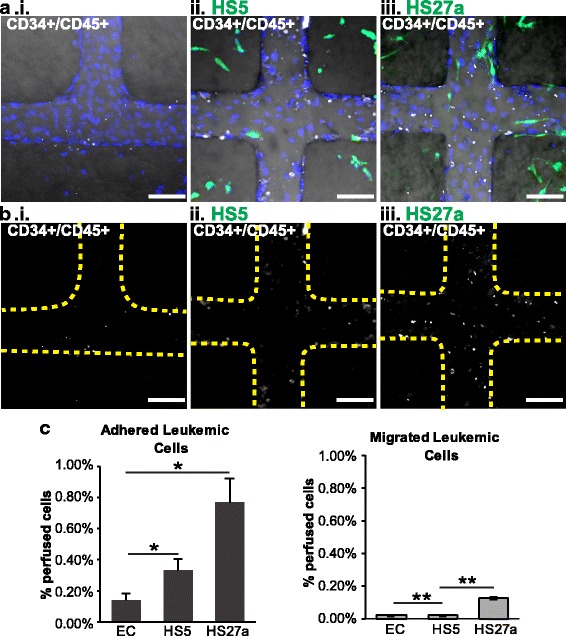
Leukemic cells perfused alone respond to microenvironmental cues. **a** Leukemic CD34^+^ cells perfused through unmodified, HS5-, and HS27a-modified vessels adhere and migrate. **b** Visualization of the leukemic cells with regards to the vessel walls (yellow dotted lines). Blue = nuclei, scale bars = 100 μm. **c** Quantification of adhesion and migration of leukemic cells. Significant differences are shown between unmodified (endothelial cell; EC) and stromal conditions unless marked. *p < 0.05, **p < 0.01

The establishment of a fibroblast niche further enables the examination of leukemic cell behavior within the *in vitro* marrow space. The differences between leukemic cells and healthy HSPCs with respect to their interactions with the endothelium are not well understood. In order to examine the affinity of leukemic cells in the fibroblast-directed microenvironments presented here, patient-derived acute myeloid leukemic cells were perfused through unmodified, HS5, or HS27a co-cultured vessels (Fig. 6). The adhesion and migration of leukemic cells was significantly increased in HS27a co-cultured vessels, with 0.77 ± 0.14% of perfused cells adhered in HS27a vessels compared with 0.14 ± 0.04% and 0.33 ± 0.07% of cells perfused through EC-only and HS5 co-cultured vessels, respectively (Fig. 6). A smaller but significant increase in adhesion was also present in leukemia cells perfused in HS5 co-cultured vessels over EC-only vessels (Fig. 6c). The same trend was present in migrated cells, with significant increases from EC-only vessels (0.01 ± < 0.01%) to HS5 vessels (0.02 ± < 0.01%) and from HS5 vessels to HS27a vessels (0.19 ± 0.04%) (Fig. 6c). Unlike HSPCs perfused alone, patient-derived CD34^+^ leukemic cells showed a strong fibroblast-directed adhesion and migration pattern (Fig. 6c).

**Fig. 7:**
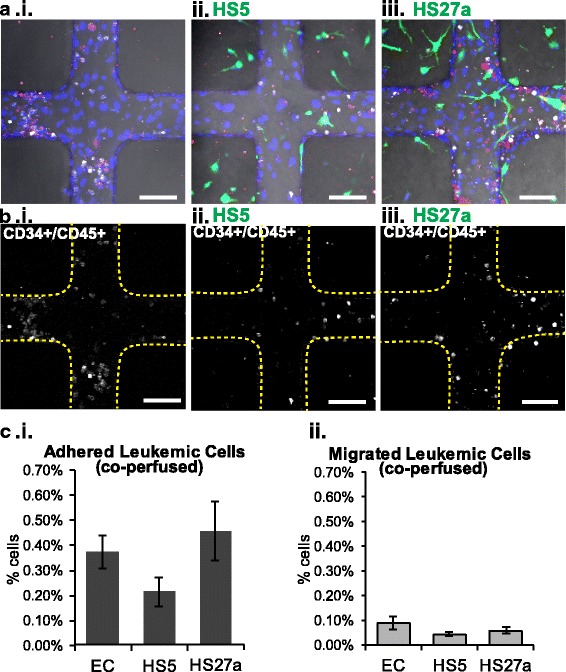
Leukemic cells perfused after monocytes do not adhere based on stromal cues. **a** Leukemic CD34^+^ cells perfused after monocytes in unmodified, HS5-, and HS27a-modified vessels. Blue = nuclei. **b** Adhesion and migration of leukemic cells are shown in relation to the vessel walls (yellow dotted lines). Scale bars = 100 μm. **c** Quantification of monocyte and leukemic CD34^+^ cell adhesion and migration. *p < 0.05. EC endothelial cell

When leukemic cells are perfused after monocytes, the distribution of leukemic cells is changed compared with perfusion of either cell type alone (Fig. 7). Co-perfused leukemic cells showed no differences in adhesion between the vessel conditions (0.37 ± 0.06%, 0.21 ± 0.05%, and 0.45 ± 0.01% in EC-only, HS5, and HS27a vessels, respectively; Fig. 7c). Transmigration of leukemic cells also showed no significant differences among vessel conditions (0.09 ± 0.02%, 0.04 ± < 0.01%, and 0.05 ± 0.01% in EC, HS5, and HS27a vessels, respectively; Fig. 7c). In combination, this suggests that when monocytes are present, patient-derived leukemic cells lose their responsiveness to fibroblast-specific environments. This system thus differentiates the niche components responsible for dictating patterns of cell adhesion and extravasation.

## Discussion

The vascular microenvironment plays an integral role in hematopoietic cell adhesion, transmigration, and engraftment [1, 7, 30, 35, 37, 65, 66]. Detailed exploration of the dynamics between niche components and the contribution of the fibroblasts, endothelial, and hematopoietic cells is needed to understand marrow function and tissue regeneration [7, 67]. Here, we have utilized an engineered microvascular platform to show that fibroblast-directed crosstalk alters hematopoietic cell adhesion and transmigration into the extravascular space.

Through the use of this multicellular co-culture with a perfusable vascular network, we first demonstrated the influence of specific marrow fibroblasts on the endothelium which subsequently influences monocyte adhesion and extravasation. HS27a and HS5 represent functionally distinct marrow components [51, 68]. In two-dimensional cultures, analysis of multicellular interactions with these cells is limited due to overgrowth. However, fibroblasts in 3D collagen are relatively nonmitotic, more closely approximating their *in vivo* behavior [67, 68]. In our system, both MSCs and HS27a fibroblasts wrapped around the vessel wall while the HS5 fibroblasts did not. The co-cultured vessels displayed different RNA expression, and the interaction of these cells with the endothelium creates a fibroblast-defined vascular niche. Though the use of marrow sinusoidal endothelial cells would be ideal, the availability of this cell type is limited. Here, we show that HUVECs are able to adapt in response to microenvironmental influences from stromal fibroblasts. Functional evidence of these changes is shown through the differential adhesion of monocytes in the fibroblast co-cultured vessels.

Our data further show that the crosstalk between circulating monocytes and fibroblasts modifies the vascular microenvironment. Perfused HSPCs showed no preferential adhesion or extravasation among any co-culture conditions. However, after the perfusion of monocytes, HSPCs demonstrated preferential recruitment into HS27a co-cultured vascular space. Leukemic CD34^+^ cells, in contrast, had the opposite trend compared with the healthy HSPCs. Alone, leukemic cells showed preferential migration towards the HS27a co-cultures. Monocyte perfusion removed the leukemic cell preference towards a fibroblast-modified microenvironment. The ability of these cells to sense and respond to differences in the vascular microenvironment demonstrates the necessity of a specific co-culture system to study hematopoietic recruitment and the niche space.

Previous studies have identified that monocytes/macrophages create a permissive niche for HSPC residence in the marrow, such that the combination of these cells with stromal fibroblasts are necessary to maintain marrow HSPC populations [3, 4, 10, 15, 46, 69]. Thus, these results suggest that the monocytes recruited to the extravascular space modulate HSPC and leukemic cell adhesion and extravasation through cellular crosstalk [50, 64, 69]. Healthy HSPCs, therefore, rely on monocytes to regulate their extravasation in the presence of stromal co-cultures, but not solely through VCAM-1-mediated adhesive interactions However, monocytes block leukemic cell sensitivity to stromal contexts, perhaps due to a loss of adhesive integrin interactions or prevention from adhesive interactions by monocytes that occupy the same binding sites. *In vivo* studies also suggest opposing niche spaces for leukemic and HSPC cells, indicating that different components are required to support leukemic or healthy niche spaces [70, 71]. The use of three separate acute myeloid leukemia patient samples in this study could have contributed to a wide variation in leukemic cell behavior. Overall, the functional crosstalk between hematopoietic cells and the vascular microenvironment is evident in this platform. Improved microphysiological models for human marrow can greatly mitigate challenges to examining multicellular interactions in hematopoietic biology.

## Conclusions

Here, we have shown that different vascular microenvironments created by functionally divergent fibroblast cell types affect hematopoietic trafficking across the vasculature. Understanding these multifaceted cellular interactions within a vascular system provides insight into the endothelial niche. In disease contexts, microenvironmental aberrations have been implicated in the induction of disease phenotypes, particularly in leukemia and other hematopoietic malignancies [70, 72–74]. There is significant potential for a tunable system such as this to be used as a tool in preclinical therapeutics testing and precision medicine. With this platform, it is possible to study in further detail the mechanisms behind dynamic spatial and temporal cell-cell interactions within the vascular niche in both healthy and disease-remodeled marrow spaces.

## Additional files

Additional file 1: **Table S1.** Primer information for RT-PCR analysis. (PDF 293 kb)

Additional file 2: **Figure S1.** Expression of CD146 by HS27a, HS5, and MSCs is shown via RT-PCR. MSC expression of CD146 is variable compared to the HS27a and HS5 cell lines. (PDF 445 kb)

Additional file 3: **Figure S2.** Immunofluorescence staining of von Willebrand Factor in an EC-only vessel after 6 days of culture. Scale bar = 50 μm. (PDF 1873 kb)

Additional file 4: **Figure S3.** RT-PCR of endothelial and stromal cells from microvessels. RT-PCR shows similar expression of CXCR4, CXCL12, E-selectin, ICAM-1, FLT-3, angiopoietin-1, IL-6, DKK3, MCP-1, HIF-1a, IL-1b, TFGb, MIP1, and GM-CSF, IL-1a (normalized to L32 ribosomal protein). KDR, P-selectin, angiopoeitin2, and FLT4 have increased expression in the endothelial-only vessels. IL-6, IL-1b, and IL-1a have increased expression in the HS5 co-cultured vessels. *p < 0.05, **p < 0.01, *** *p* < 0.001, ****p < 0.0001. (PDF 2015 kb)

Additional file 5: **Figure S4.** Monocyte adhesion in HS27a vessels. (A) Monocytes perfused through EC, EC with HS27a-conditioned media, or HS27a co-cultured vessels. (B) Quantification of monocyte adhesion shows no changes in adhesion between EC-only and EC with HS27a-conditioned media but an increase within the HS27a co-cultured vessels. Scale bars = 100 μm. (PDF 858 kb)

Additional file 6: **Figure S5.** Expression of VCAM-1 in monocytes co-cultured with stromal fibroblasts and conditioned media. Microarray expression analysis of (A) monocytes from two different donors alone. (B) Expression of VCAM in HS5 cells, monocytes cultured with HS5-conditioned media, and monocytes co-cultured with HS5 cells. (C) Expression of VCAM in HS27a cells, monocytes cultured with HS27a-conditioned media, and monocytes co-cultured with HS27a cells. Expression values extracted from microarray data from Iwata et al. [44] (http://www.ncbi.nlm.nih.gov/geo/; accession numbers GSE9390 and GSE10595, gene ID: 203868_s_at) (PDF 152 kb)

Additional file 7: **Figure S6.** Monocytes, not VCAM-1, determine HSPC trafficking in HS27a vessels. (A) HSPCs were perfused through HS27a co-cultured vessels (i) alone, (ii) after monocyte perfusion, or (iii) after monocyte and VCAM-1 blocking antibody perfusion. (B) HSPCs are shown with the vessel boundary (yellow dotted line). Scale bars = 100 μm. Quantification of (C) HSPC adhesion and (D) migration behavior from these vessels show that monocytes change HSPC adhesion and migration but blocking VCAM-1 in the presence of monocytes does not significantly change adhesion and migration. *p < 0.05, **p < 0.01, *** *p* < 0.001. (PDF 889 kb)
