# Supplementary material for: Engineering a multicellular vascular niche to model hematopoietic cell trafficking
Source: Stem Cell Res Ther. 2018 Mar 23;9:77. doi: 10.1186/s13287-018-0808-2 (PMC5865379; doi:10.1186/s13287-018-0808-2)
Supplement: Supplementary file 1 — Table S1. Primer information for RT-PCR analysis. (PDF 293 kb) [file 13287_2018_808_MOESM1_ESM.pdf]

Table S1.

Table S1. Primer information for RT-PCR analysis.

Primers used for RT-PCR	Sequence
L32 – for normalization	AGTTCCTGGTCCACAACGTC
	TTGGGGTTGGTGACTCTGAT
cMYC ex3 /hMYC Ex3	AAGGACTATCCTGCTGCCAA
	CGCCTCTTGACATTCTCCTC
ICAM1 ex7	GTACCTCTATAACCGCCAGC
	GCTTGTGTGTTCGGTTTCAT
VCAM1 ex9	ATGCCCATCTATGTCCCTTG
	GCAGTTACTGTTCTTCAGGC
MCP1 ex3	AAACCCAAACTCCGAAGACT
	ATAAAACAGGGTGTCTGGGG
TEK ex23	AGCCAGTCCCGTTTCATTTA
	TGTTAGCATCCTGGACTGAC
VWF ex52	ACCAATGGCTCTGTTGTGTA
	CTCACTTGCTGCACTTCCT
PECAM1 ex2	ACAACCCAGTAGAGATGCAG
	ATTTTCCACCATGACGCTTG
KDR ex 30-1	GCCCCATCCTCAAAGAAGTA
	CGAACACTTACATTGCCTGG
Angpt1 ex9	TCTGCCTGTAAGTGTCCAAG
	GGAGGCCCAGTAGCTTTATT
Angpt2 Ex9	TCTTATGAGCGAGAATGGGG
	AAGCTCCTCTCAGCAAAACT
cFOS ex4	TTCCTCTGACTCGCTCAGCTC
	AGTCCTTGAGGCCCACAGC
Egr1 ex2-2	ACTCCTCTGTTCCCCCTGCT
	GTCCTGGGAGAAAAGGTTGCT
CXCR4 ex2	GGCCAAGTTCTTAGTTGCTG
	GATTCACTACACGCTCTGGA
CXCL12 ex4	TCTGTGAGATCCGTCTTTGG
	AGACTTGTCTTTTGCGGGTA
FLT3 ex24	GGATTTGGGGCTACTCTCTC
	TAGGGATAGGTGGAGGGATG
FLT4 ex30	CGGTCTGGTTCTTCCCTTTA
	TCTCTCATGAGCTGGTTCAC
IL6 ex5	GGCACCTCAGATTGTTGTTG
	CATAAGTTCTGTGCCCAGTG
IL8 ex4	GTGTTGGTAGTGCTGTGTTG
	ATTGACTGTGGAGTTTTGGC
NFKB ex24	GCAAACTCAGCTTTACCGAG
	GTCCTTCCTGCCCATAATCA
RELA ex11	AGCCCACAAAGCCTTATCAA
	CTGGTATCTGGGGCGTTATT
DKK3 ex14	CCCTGTCCAGATTATTGGCT
	CATCTCCTCCCCTCAAACAA
SELE ex4	AGCAAAGGTGAAGAGACCAA
	TGTGCCAAGATTTTACAGCG

SupplementaryTable 1. (Continued)

Primers used for RT-PCR	Sequence
CSF2 Ex4	TTCCTGTGCAACCCAGATTA
	AAGGGGATGACAAGCAGAAA
HIF1a ex15	TTAGCATGTAGACTGCTGGG
	GCTGCCTTGTATAGGAGCAT
IL1a ex7	CCATCACAGGTAGTGAGACC
	AACAAGTTTGGATGGGCAAC
IL1b ex7	AGAGAGTCCTGTGCTGAATG
	AGACAACAGGAAAGTCCAGG
IL3 ex5	CGGAGGAAACTGACGTTCTA
	TGGACGTTGGACTCAAAAGA
IL1 ex4	GAAGGAAGCCAACCAGAGTA
	TCAGCTCGAACACTTTGAAT
TGFb ex7	ATGGGGGCTGTATTTAAGGA
	GCAGTCCTCTCTCCATCTTT
TGBS1 ex22	TCAAACCAGTGTAAGGCAGT
	TTCCTGCTACATCTGCACAA
TIMP1 ex6	CCCAGATAGCCTGAATCCTG
	AAAGATGGGAGTGGGAACAG
TIMP3 ex5	TGAGCCCAGACTTGATGTTT
	AGGGAACTTGTGTAGGTTGG
CHRDL1 ex12	TCAGCCAGATGTGTTCAAGT
	GCCCTTTTCAGATCTCTCCA
KITLG ex10	GCTTTGCACCTCTTTGGTAG
	TCACTCAGGAGGCAACATTT
MIP1a ex3	AAGCCACCAGACTGACAAAT
	TTATTATTTCCCCAGGCCGA
Notch1 ex34	TCCACCAGTTTGAATGGTCA
	AGAGGGTTGTATTGGTTCGG
OCLN ex9	ACAGCAATGGAAAACCACAC
	CCAAAGGAATGGGAAACGAC
PDGFb ex7	CGTTTTGAAGACGTGGACTC
	ACTCCATCTCTAACCCACCT
ROBO4 ex18	TGCCAAACAAAGGGTTCAAG
	TGACAACAGTACGAGGATGG
SELP ex17	CAGTGGTTGCTGTTGATGAG
	CTTGATTCTTGGCCTTCTGC
CDH5 ex5	GCCAATCCATGCTCTCTTTC
	TAAAGGCCACATCTTGGGTT
CSPG4 ex10	ACCAGGGTAACCTCCTACAT
	CCTTCTCCTTGCCCTCTTAG
FLT1 ex30	TAAGGTCAAGGGAAAACCCC
	AAACGTGACTGACTTCCTGT
