# Supplementary material for: Engineering a multicellular vascular niche to model hematopoietic cell trafficking
Source: Stem Cell Res Ther. 2018 Mar 23;9:77. doi: 10.1186/s13287-018-0808-2 (PMC5865379; doi:10.1186/s13287-018-0808-2)
Supplement: Supplementary file 2 — Figure S1. Expression of CD146 by HS27a, HS5, and MSCs is shown via RT-PCR. MSC expression of CD146 is variable compared to the HS27a and HS5 cell lines. (PDF 445 kb) [file 13287_2018_808_MOESM2_ESM.pdf]

Figure S1.

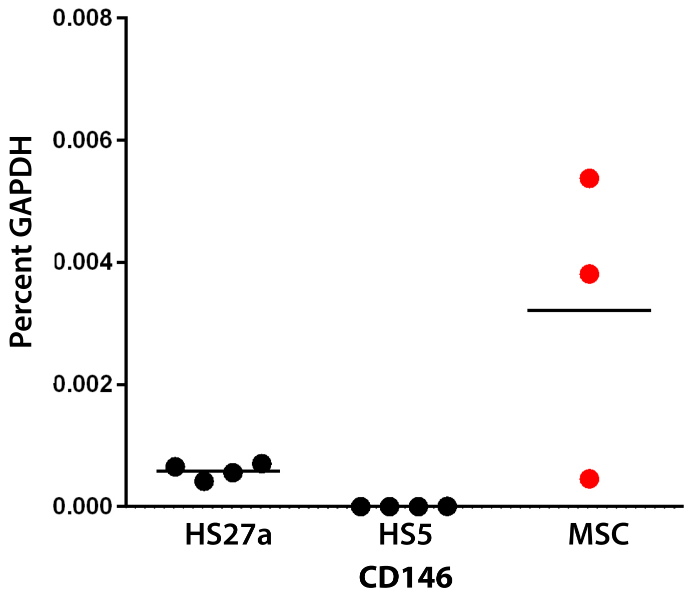

Figure S1. Expression of CD146 by HS27a, HS5, and MSCs is shown via RT-PCR. MSC expression of CD146 is variable compared to the HS27a and HS5 cell lines.
