# Supplementary material for: Engineering a multicellular vascular niche to model hematopoietic cell trafficking
Source: Stem Cell Res Ther. 2018 Mar 23;9:77. doi: 10.1186/s13287-018-0808-2 (PMC5865379; doi:10.1186/s13287-018-0808-2)
Supplement: Supplementary file 3 — Figure S2. Immunofluorescence staining of von Willebrand Factor in an EC-only vessel after 6 days of culture. Scale bar = 50 μm. (PDF 1873 kb) [file 13287_2018_808_MOESM3_ESM.pdf]

Supplementary Figure 3.

vWF Nuclei

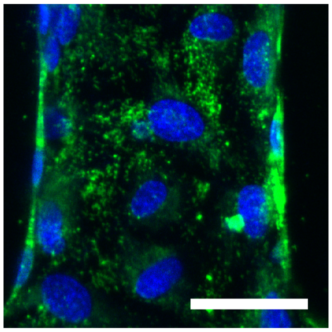

Supplementary Figure 3. Immunofluorescence staining of von Willebrand Factor in an EC only vessel after 6 days of culture. Scale bar = 50 μm.
