# Supplementary material for: Engineering a multicellular vascular niche to model hematopoietic cell trafficking
Source: Stem Cell Res Ther. 2018 Mar 23;9:77. doi: 10.1186/s13287-018-0808-2 (PMC5865379; doi:10.1186/s13287-018-0808-2)
Supplement: Supplementary file 4 — Figure S3. RT-PCR of endothelial and stromal cells from microvessels. RT-PCR shows similar expression of CXCR4, CXCL12, E-selectin, ICAM-1, FLT-3, angiopoietin-1, IL-6, DKK3, MCP-1, HIF-1a, IL-1b, TFGb, MIP1, and GM-CSF, IL-1a (normalized to L32 ribosomal protein). KDR, P-selectin, angiopoeitin2, and FLT4 have increased expression in the endothelial-only vessels. IL-6, IL-1b, and IL-1a have increased expression in the HS5 co-cultured vessels. *p < 0.05, **p < 0.01, *** p < 0.001, ****p < 0.0001. (PDF 2015 kb) [file 13287_2018_808_MOESM4_ESM.pdf]

Figure S3.

vWF Nuclei

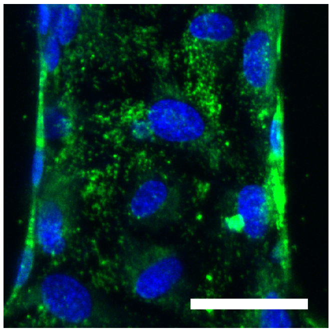

Figure S3. Immunofluorescence staining of von WIllebrand Factor in an EC only vessel after 6 days of culture. Scale bar = 50 μm.
