# Supplementary material for: Engineering a multicellular vascular niche to model hematopoietic cell trafficking
Source: Stem Cell Res Ther. 2018 Mar 23;9:77. doi: 10.1186/s13287-018-0808-2 (PMC5865379; doi:10.1186/s13287-018-0808-2)
Supplement: Supplementary file 5 — Figure S4. Monocyte adhesion in HS27a vessels. (A) Monocytes perfused through EC, EC with HS27a-conditioned media, or HS27a co-cultured vessels. (B) Quantification of monocyte adhesion shows no changes in adhesion between EC-only and EC with HS27a-conditioned media but an increase within the HS27a co-cultured vessels. Scale bars = 100 μm. (PDF 858 kb) [file 13287_2018_808_MOESM5_ESM.pdf]

Figure S4.

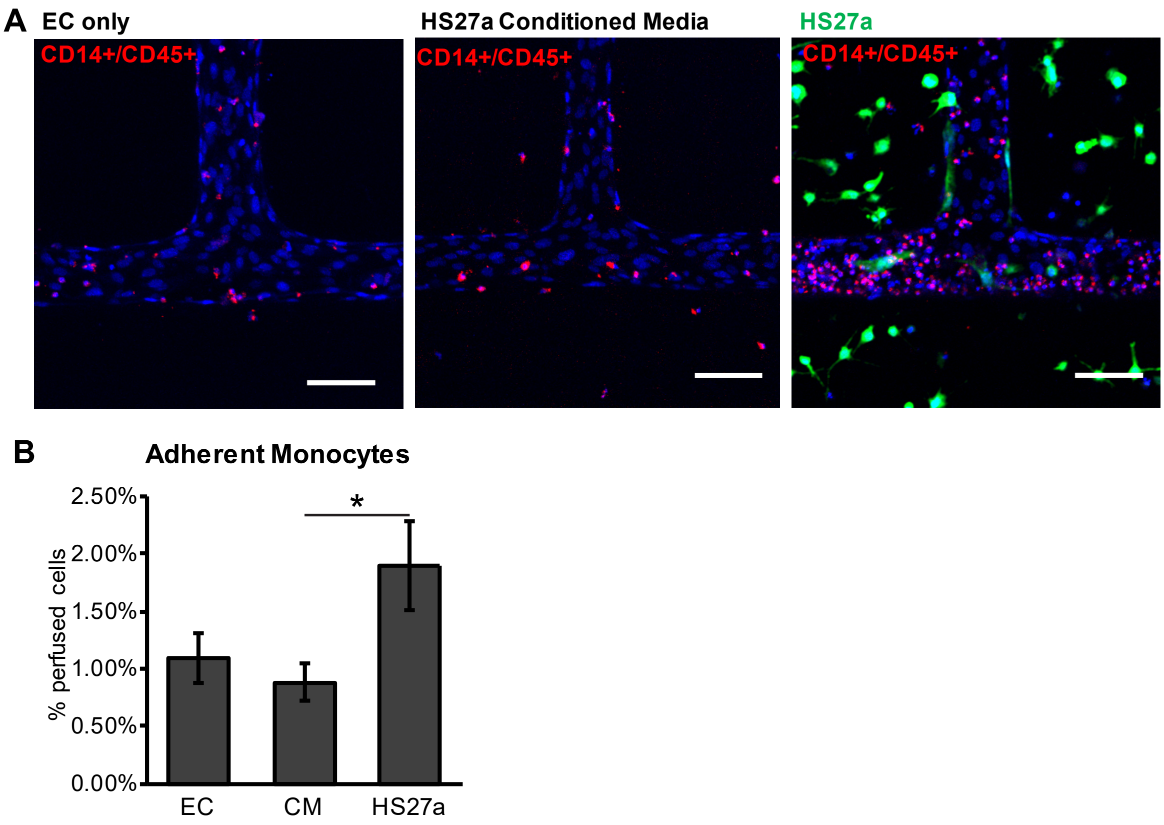

Figure S4. **Monocyte adhesion in HS27a vessels.** (A) Monocytes perfused through EC, EC with HS27a-conditioned media, or HS27a-co-cultured vessels. (B) Quantification of monocyte adhesion shows no changes in adhesion between EC only and EC with HS27a-conditioned media but an increase within the HS27a-co-cultured vessels. Scale bars = 100 µm.
