# Supplementary material for: Engineering a multicellular vascular niche to model hematopoietic cell trafficking
Source: Stem Cell Res Ther. 2018 Mar 23;9:77. doi: 10.1186/s13287-018-0808-2 (PMC5865379; doi:10.1186/s13287-018-0808-2)
Supplement: Supplementary file 6 — Figure S5. Expression of VCAM-1 in monocytes co-cultured with stromal fibroblasts and conditioned media. Microarray expression analysis of (A) monocytes from two different donors alone. (B) Expression of VCAM in HS5 cells, monocytes cultured with HS5-conditioned media, and monocytes co-cultured with HS5 cells. (C) Expression of VCAM in HS27a cells, monocytes cultured with HS27a-conditioned media, and monocytes co-cultured with HS27a cells. Expression values extracted from microarray data from Iwata et al. [44] (http://www.ncbi.nlm.nih.gov/geo/; accession numbers GSE9390 and GSE10595, gene ID: 203868_s_at) (PDF 152 kb) [file 13287_2018_808_MOESM6_ESM.pdf]

Supplementary Figure 5.

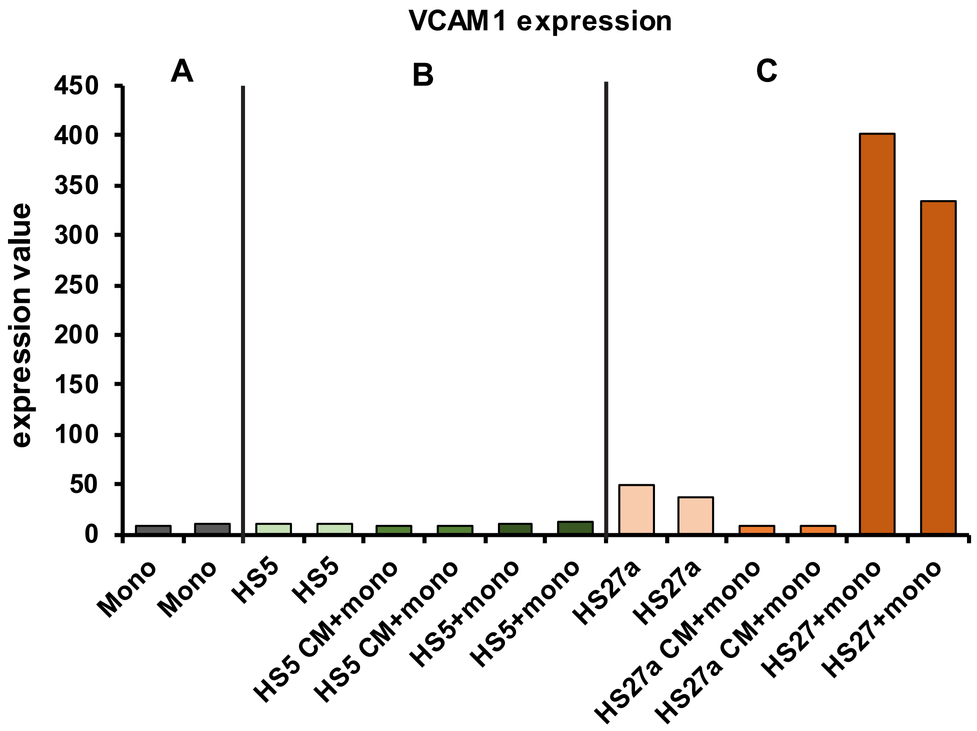

Supplementary Figure 5. Expression of VCAM1 in monocytes co-cultured with stromal fibroblasts and conditioned media. Microarray expression analysis of (A) monocytes from two different donors alone. (B) Expression of VCAM in HS5 cells, Monocytes cultured with HS5 conditioned media, and Monocytes co-cultured with HS5 cells. (C) Expression of VCAM in HS27a cells, Monocytes cultured with HS27a conditioned media, and Monocytes co-cultured with HS27a cells. Expression values extracted from microarray data from Iwata et al. [44] (http://www.ncbi.nlm.nih.gov/geo/; accession numbers GSE9390 and GSE10595, gene ID: 203868_s_at)
