# Supplementary material for: Engineering a multicellular vascular niche to model hematopoietic cell trafficking
Source: Stem Cell Res Ther. 2018 Mar 23;9:77. doi: 10.1186/s13287-018-0808-2 (PMC5865379; doi:10.1186/s13287-018-0808-2)
Supplement: Supplementary file 7 — Figure S6. Monocytes, not VCAM-1, determine HSPC trafficking in HS27a vessels. (A) HSPCs were perfused through HS27a co-cultured vessels (i) alone, (ii) after monocyte perfusion, or (iii) after monocyte and VCAM-1 blocking antibody perfusion. (B) HSPCs are shown with the vessel boundary (yellow dotted line). Scale bars = 100 μm. Quantification of (C) HSPC adhesion and (D) migration behavior from these vessels show that monocytes change HSPC adhesion and migration but blocking VCAM-1 in the presence of monocytes does not significantly change adhesion and migration. *p < 0.05, **p < 0.01, *** p < 0.001. (PDF 889 kb) [file 13287_2018_808_MOESM7_ESM.pdf]

Figure S6.

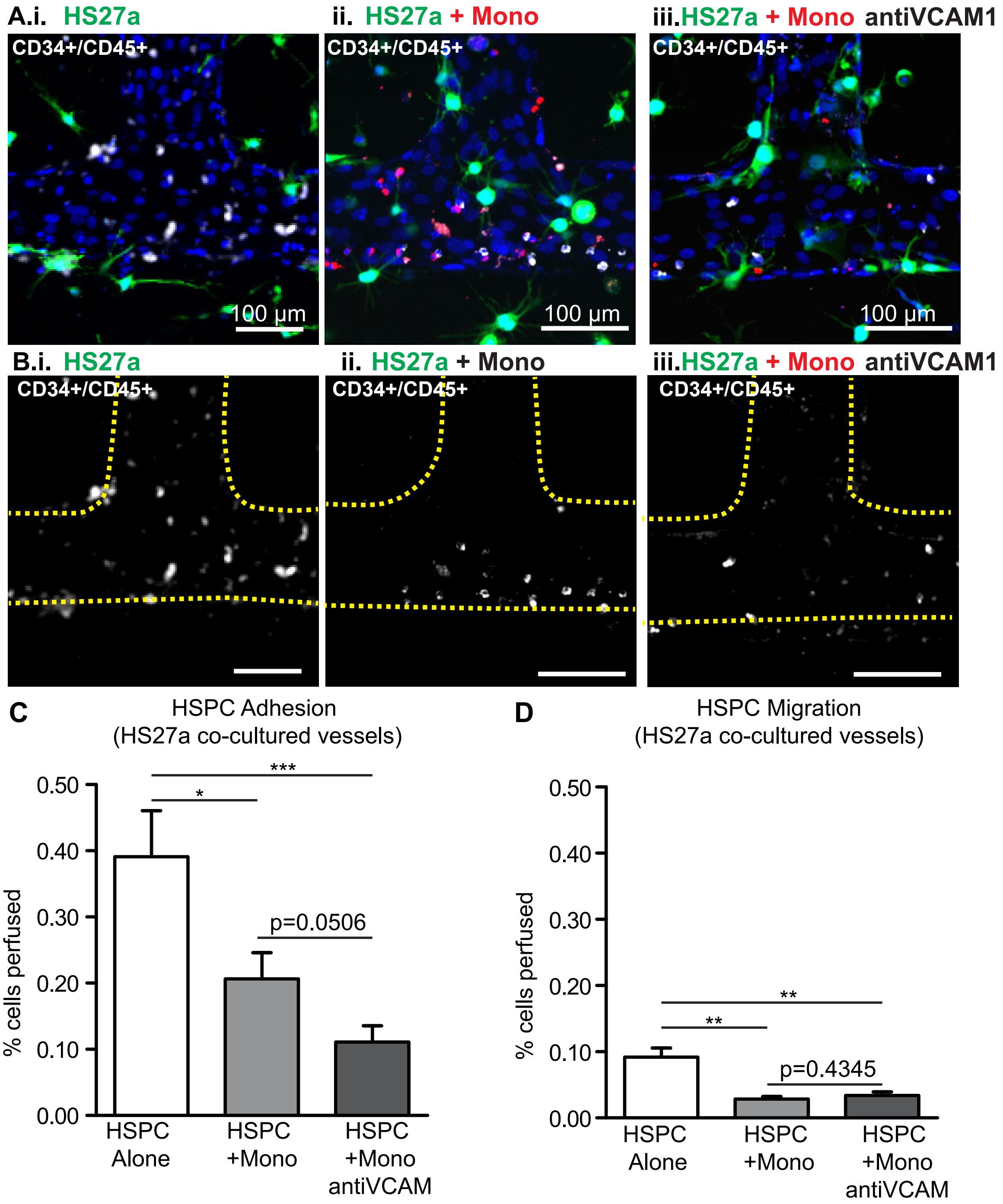

Figure S6. Monocytes, not VCAM1, determine HSPC trafficking in HS27a vessels. (A) HSPCs were perfused through HS27a co-cultured vessels (i) alone, (ii) after monocyte perfusion, or (iii) after monocyte and VCAM1 blocking antibody perfusion. (B) HSPCs are shown with the vessel boundary (yellow dotted line). Scale bars: 100 µm. Quantification of (C) HSPC adhesion and (D) migration behavior from these vessels show that monocytes change HSPC adhesion and migration but blocking VCAM1 in the presence of monocytes does not significantly change adhesion and migration. $^{*}p < 0.05$, $^{**}p < 0.01$, $^{***}p < 0.001$.
